# Financial performance of rural and urban nursing homes: A comparative analysis

**DOI:** 10.1111/jrh.70053

**Published:** 2025-07-22

**Authors:** Gregory N. Orewa, Rohit Pradhan, Akbar Ghiasi, Shivani Gupta, Robert Weech‐Maldonado

**Affiliations:** ^1^ Department of Public Health & Department of Management, College of Health, Community, and Policy & Carlos Alvarez College of Business University of Texas at San Antonio San Antonio Texas USA; ^2^ School of Health Administration Texas State University San Marcos Texas USA; ^3^ H‐E‐B School of Business and Administration University of Incarnate Word San Antonio Texas USA; ^4^ College of Business University of Houston Clear Lake Houston Texas USA; ^5^ Department of Health Services Administration, School of Health Professions University of Alabama Birmingham Birmingham Alabama USA

**Keywords:** geography, health care financing, health disparities, long‐term care, policy

## Abstract

**Purpose:**

The financial sustainability of nursing homes is increasingly critical as the aging US population continues to grow. Rural facilities often encounter more significant economic challenges than urban counterparts. This study investigates the disparities in financial performance between rural and urban nursing homes in the United States, emphasizing the influence of organizational and environmental factors. A comprehensive understanding of these differences is necessary for the implementation of effective policy and management interventions.

**Methods:**

The study used a longitudinal dataset (2018–2022) comprising 66,056 nursing home‐year observations. Data sources included Centers for Medicare and Medicaid Services (CMS) Cost Reports, Payroll‐Based Journal, Care Compare, LTCFocus, and the Area Health Resource File. The dependent variable was the operating margin. The primary independent variable, geographic location, was classified using Rural–Urban Commuting Area (RUCA) codes. We conducted multivariable linear regression with facility‐level random effects and two‐way fixed effects (state and year) to assess rural–urban financial disparities while controlling for organizational and environmental factors and the impact of COVID‐19.

**Findings:**

Rural nursing homes had lower operating margins than urban facilities in unadjusted models. However, after adjusting for organizational factors such as size, occupancy, and payer mix, the rural–urban difference was no longer significant. Environmental factors, including population demographics and income levels, contributed to financial disparities. COVID‐19 exacerbated financial challenges, disproportionately affecting rural facilities.

**Conclusions:**

Financial disparities between rural and urban nursing homes are not solely due to geographical location, but also stem from structural challenges. These insights have significant policy implications suggesting that addressing reimbursement rates, operational efficiency, and resource allocation is crucial to ensure the financial sustainability and quality care for aging populations.

## INTRODUCTION

The growing number of older adults in the United States represents a significant demographic shift. Research indicates that nursing homes, particularly those in underserved areas, may struggle to meet the growing demand for high‐quality care without strategic policy interventions and sustainable funding models.^1^ Each year, over four million Americans either enter or reside in nursing homes, underscoring their critical role in the national health care landscape.^2^ However, despite this burgeoning need, many nursing homes, particularly those in underserved areas, face declining occupancy rates, which may exacerbate existing financial challenges. Financially, nursing homes often also face a decline in care quality and operational sustainability.^3^, ^4^

Facilities in both rural and urban areas must navigate capacity limitations, resource constraints, competition, and evolving health care models.^5^, ^6^ Yet, financial health may vary significantly between rural and urban facilities, further complicating the provision of consistent, quality care.^7^ Therefore, understanding rural–urban differences in nursing homes’ financial performance is important to ensure access to high‐quality long‐term care services, adequate staff, and sustainability.^3^, ^8^

Financial performance differences between rural and urban nursing homes may stem from various organizational and market factors that influence the demand for and availability of critical resources.^7^, ^9^, ^10^ Organizational resources, such as payer mix, represent the inputs facilities require to deliver care.^11^, ^12^ Rural facilities often have fewer resources compared to urban facilities.^13^, ^14^ Beyond internal resources, environmental conditions can also shape financial differences between urban and rural nursing homes. For example, compared to rural nursing homes, urban facilities face more intense competition from other facilities and alternative care providers, such as assisted living facilities, which may push them to adopt advanced care models or diversify service offerings to remain financially viable.^5^, ^15^

Several studies have examined the factors contributing to rural–urban variations in nursing home operations. For instance, rural nursing homes often face challenges in recruiting and retaining qualified personnel, resulting in greater use of agency staffing and higher recruitment and retention costs.^10^, ^16^ Additionally, rural nursing homes may be more reliant on government funding to maintain operations, as they have fewer private‐pay residents.^11^, ^17^, ^18^ The COVID‐19 pandemic further exposed these disparities, as rural facilities faced disproportionate challenges due to limited financial resources, staff shortages, and rising personal protective equipment (PPE) costs.^19^, ^20^, ^21^ Urban facilities fared better during the same period because they had better access to federal assistance and diverse payer mixes, and they benefited from economies of scale.^19^, ^22^

Despite extensive research on organizational and market dynamics affecting operational differences between rural and urban nursing homes, there has been limited focus on financial performance differences.^2^, ^3^ Smith et al. provided an early comparison of financial performance, organizational characteristics, and management strategies between rural and urban facilities over 30 years ago.^7^ However, given the significant transformations in policy,^19^, ^23^ mergers and acquisitions,^24^, ^25^, ^26^ private equity dominance,^25^, ^27^ and reimbursement mechanisms over the past three decades,^23^, ^28^, ^29^ updated research in this domain is needed.

The primary goal of this study was to examine the differences in financial performance between urban and rural nursing homes. Furthermore, we also examined the organizational‐ and environmental‐level factors that may explain these differences. By highlighting the factors that influence financial outcomes in these different contexts, this study aims to provide insights that can inform policy and management practices, ultimately contributing to the strategic relevance and sustainability of nursing homes across different geographic settings. Understanding the distinct financial landscapes of rural and urban nursing homes is crucial for developing targeted strategies that can enhance nursing homes' financial health and ensure the sustainability of nursing home care across the country.

## METHODS

### Data sources

**FIGURE 1:**
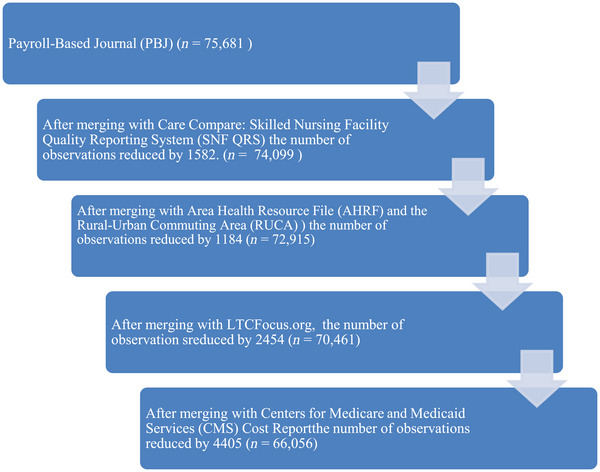
Data merge steps.

The following secondary datasets were used to conduct this study: Payroll‐Based Journal (PBJ), Care Compare: Skilled Nursing Facility Quality Reporting System (SNF QRS), LTCFocus, Centers for Medicare and Medicaid Services (CMS) Cost Report, Area Health Resource File (AHRF), and the Rural–Urban Commuting Area (RUCA) codes for the period 2018–2022. The PBJ provides detailed auditable data on nursing home staffing hours. SNF QRS captures nursing home quality data (star rating). LTCFocus provides facility and environmental characteristics. CMS Cost Report was used to calculate financial performance. AHRF contains county‐level sociodemographic data. The Medicare identification number was used to merge the different datasets, except AHRF and the RUCA codes, which were merged using the Federal Information Processing System (FIPS) code. The final analytic data file comprised 66,056 nursing home‐year observations with an average of 13,211 unique facilities per year. Figure 1 outlines the data merge steps and construction of the final analytic sample.

#### Variables

##### Dependent variable

Profitability is an important metric since it reflects efficiency with which a firm turns its business activities into profits.^30^ In our study, we measure profitability utilizing operating margin, which is a commonly used financial performance measure in the nursing home literature.^6^, ^19^ We selected operating margin over total margin because it more accurately captures the core business functions and operational efficiency of nursing homes. Unlike total margin, which includes nonoperating income such as endowments and nonoperating expenses like interest payments, operating margin isolates income and expenses related to resident care and day‐to‐day operations.^31^ While total margin provides a broader view of overall profitability, it can be significantly affected by external funding sources or nonpatient revenues. These influences may obscure the relationship between organizational or structural characteristics and financial performance derived from routine operations. We calculated the operating margin as (Net Patient Revenue − Operating Costs)/Net Patient Revenue.

##### Independent variable

The primary independent variable was the geographic location of nursing homes in terms of rural or urban. This classification was operationalized using the RUCA codes, which categorize areas based on population density, urbanization, and daily commuting patterns. Specifically, nursing homes located in areas designated as micropolitan (RUCA codes 4–6), small town (RUCA codes 7–9), and rural (RUCA code 10) were collectively classified as “rural” and coded as 0. Conversely, facilities in metropolitan areas (RUCA codes 1–3) were classified as “urban” and coded as 1. This definition aligns with the rural definition set forth by the United States (US) Health Services & Resource Administration (HRSA).^32^

##### Control variables

We also included facility‐level and environmental‐level characteristics of a nursing home that may explain the differences in financial performance between urban and rural nursing homes.^6^, ^19^ Facility‐level variables include the following: nursing home quality as indicated by the star rating system, which ranges from 1 to 5 stars, with higher rating reflecting better quality; nursing staff (registered nurse [RN], license practical nurse [LPN], and certified nurse assistant [CNA]) hours per resident day (PRD); chain affiliation; ownership status (for‐profit, not‐for‐profit, and government); occupancy rate (percentage of occupied beds); size (total number of resident currently living in a facility); nursing home payer mix (percentages of Medicare, Medicaid, and private‐pay residents); resident complexity (average acuity index or case mix score at the facility level), and race/ethnicity of the residents (White, Black, Hispanic, and other).

We also included the following nursing home environmental characteristics at the county level: percentage of the population 65 years and older; household income (pretax cash income of the householder and all other people 15 years old and older in the household, whether or not they are related to the householder); and Medicare advantage (MA) penetration (percentage of Medicare beneficiaries in MA). In addition, we account for competition, which was assessed using the Herfindahl–Hirschman index (HHI), a metric that evaluates market concentration within an industry. HHI is calculated by summing the squared value of each nursing home's market share based on inpatient days, with values ranging between 0 and 1. Competition was defined as 1 − HHI, where higher values indicate greater market competition. Finally, we included a COVID‐19 indicator (1 = 2020–2022: 0 = 2018 and 2019) to account for the effects of the pandemic on nursing home financial performance, and an interaction of rural/urban with COVID‐19 to assess the differential effect of the pandemic based on location.

#### Statistical analysis

Our unit of analysis was the nursing home. We used descriptive statistics to summarize our dependent, independent, and control variables: mean and standard deviation for continuous variables and frequency and percent for categorical variables. In addition, we conducted bivariate statistics comparing the operating margin and the organizational and market variables by rural and urban location. To quantify the magnitude of observed differences, we calculated effect sizes. Cohen's *d* was used for quantitative variables, with interpretive thresholds of small (<0.2), medium (0.2–0.5), and large (>0.6). Somers’ *D* was used to assess the ordinal association between star rating and location, with thresholds of (0.10–0.20) for weak, (0.20–0.30) for moderate, and (0.30–0.50) for strong associations. Cramér's *V* measured effect sizes for nominal variables such as chain affiliation and ownership type by location and was interpreted as very weak (0.01–0.10), weak (0.10–0.20), moderate (0.20–0.30), and strong (0.30–0.50).

We modeled the data using multivariable linear regression with facility‐level random effects and two‐way fixed effects (state and year). Random effects capture facility‐specific, time‐invariant variations, accounting for correlation among repeated observations in longitudinal data. State fixed effects account for interstate differences, while year fixed effects account for time trends. We used five nested sequential models to evaluate the separate contributions of organizational factors, environmental factors, and COVID‐19 on the rural/urban differences in operating margin.

*Model 1* analyzed the relationship between the relationship between geographic location (rural/urban) and financial performance.

*Model 2* included the variables from Model 1 plus the organizational factors.

*Model 3* included the variables from Model 1 plus the environmental factors.

*Model 4* included the variables from Model 1 plus the organizational and environmental factors.

*Model 5* included the variables from Model 4 plus the COVID‐19 indicator and an interaction term between COVID‐19 and geographic location.

We found no evidence of multicollinearity among the variables (i.e., variance inflation factor (VIF) = > 5, *r* < 0.8). Stata 16.1 was used for statistical analysis. Statistical significance was evaluated at a 0.05 or smaller alpha level.

## RESULTS

Table 1 presents the bivariate statistics showing the variation in nursing home characteristics such as operating margin, organizational‐, and environmental‐level factors based on their geographic location (rural vs. urban), and effect size. Urban nursing homes show a higher profitability, with an average operating margin of 5.3%, compared to 3.0% for rural nursing homes (*p* = 0.001). This represented a small effect size. Among organizational factors size (resident count) resident complexity and resident racial/ethnic mix (percentage of White) had a large effect size. Nursing staff hours PRD (RN, LPN, and CNA) and percentage of Medicare residents, resident racial/ethnic mix (percentage of Black, Hispanic, and other race) had a medium effect size. Small effect size was found in star rating, occupancy rates, percentage of private‐pay and Medicaid residents and resident racial/ethnic mix (percentage of Asian). While chain affiliation and ownership status showed very small effects size. Among the environmental factors, market competition (HHI), percentage of population 65+ and over, household income and MA penetration all had a large effect size.

**TABLE 1:** Descriptive table of nursing homes by location (rural vs. urban; *N* = 66,056 nursing home‐year observations).

	Rural mean (SD)/*N* (%)	Urban mean (SD)/*N* (%)	*p* value	Cohen's *d*, Somers’ *D*, and Cramér's *V*	Effect size
***Dependent variable***					
Operating margin (%)	3.02 (21.26)	5.34 (24.27)	0.001	−0.10	Small
***Organizational‐level factors***					
***Star rating***					
*	1915 (9.92)	2056 (4.47)			
**	3708 (19.21)	5639 (12.26)		0.14	Small
***	4630 (23.99)	9291 (20.2)	0.001		
****	4735 (24.53)	12,290 (26.72)			
*****	4314 (22.35)	16,719 (36.35)			
Nursing staff hours PRD					
RN	0.39 (0.26)	0.46 (0.34)	0.001	−0.23	Medium
LPN	0.75 (0.30)	0.85 (0.34)	0.001	−0.31	Medium
CNA	2.02 (0.54)	2.15 (0.59)	0.001	−0.23	Medium
Chain affiliated					
No	9287 (47.47)	20,993 (44.92)			
Yes	10,277 (52.53)	25,738 (55.08)	0.001	0.05	Very Small
Ownership status					
For‐profit	13,941 (71.26)	35,423 (75.80)			
Not‐for‐profit	3627 (18.54)	8756 (18.74)	0.001	0.08	Very Small
Government	1996 (10.20)	2552 (5.46)			
Occupancy rate (%)	72.48 (17.83)	77.94 (42.17)	0.001	−0.17	Small
Size (resident count)	65.29 (31.69)	92.22 (52.60)	0.001	−0.62	Large
Payer mix					
% of private residents	29.16 (21.37)	31.23 (23.52)	0.001	−0.09	Small
% of Medicare residents	11.87 (10.53)	14.78 (13.56)	0.001	−0.24	Medium
% Medicaid residents	58.97 (22.53)	53.98 (27.22)	0.001	0.20	Small
Resident complexity	2.3 (0.008)	2.28 (0.002)	0.001	0.75	Large
Resident racial/ethnic mix					
% White	88.67 (17.18)	75.27(24.75)	0.001	0.63	Large
% Black	3.86 (11.79)	10.62 (18.57)	0.001	−0.44	Medium
% Asian	0.79 (1.68)	1.16 (6.55)	0.001	−0.08	Small
% Hispanic	1.10 (7.57)	4.08 (11.29)	0.001	−0.31	Medium
% other race	5.85 (10.13)	8.87 (12.50)	0.001	−0.27	Medium
***Environmental‐level factors***					
Competition (1 − HHI)	0.57 (0.27)	0.88 (0.16)	0.001	−1.40	Large
Population 65 and over (%)	20.12 (3.80)	16.68 (3.87)	0.001	0.90	Large
Household income (USD)	52,408.08 (10,419.19)	68,280.25 (17,513.20)	0.001	−1.10	Large
Medicare advantage penetration (%)	30.70 (14.51)	41.22 (12.72)	0.001	−0.77	Large

Effect sizes: Cohen's *d* was used for quantitative variables, with thresholds of small (<0.2), medium (0.2–0.5), and large (>0.6). Somers’ *D* assessed the association between star rating and location (0.10–0.20 = weak, 0.20–0.30 = moderate, 0.30–0.50 = strong). Cramér's *V* measured effect sizes for nominal variables like chain affiliation and ownership with location (0.00–0.10 = very weak, 0.10–0.20 = weak, 0.20–0.30 = moderate, 0.30–0.50 = strong).

Abbreviations: CNA, certified nurse assistant; HHI, Herfindahl–Hirschman index; LPN, licensed practical nurse; PRD, per resident day; RN, registered nurse.

Table 2 presents the results of the multivariable regression. The regression analysis results are presented in five models, adjusting for various sets of covariates. Model 1, the unadjusted model, indicated that rural nursing homes had 2.19% lower operating margin compared to urban nursing homes (*β* = −2.19; 95% confidence interval [CI], −3.77 to −1.01; *p* < 0.01). In Model 2, after accounting for organizational factors, the association between rural location and operating margin was no longer statistically significant (*β* = −0.72; 95% CI, −2.46 to 1.01; *p* > 0.05). Compared to 1‐star rated facilities, nursing homes with higher CMS star ratings had progressively higher operating margins. Specifically, 2‐star (*β* = 0.73; 95% CI, 0.22–1.23; *p* < 0.01), 3‐star (*β* = 1.41; 95% CI, 0.89–1.92; *p* < 0.001), 4‐star (*β* = 1.80; 95% CI, 1.27–2.33; *p* < 0.001), and 5‐star facilities (*β* = 2.15; 95% CI, 1.59–2.70; *p* < 0.001) all demonstrated significantly better financial performance. Chain‐affiliated nursing homes had significantly higher operating margins compared to nonchain facilities (*β* = 1.68; 95% CI, 1.32–2.03; *p* < 0.001). Staffing levels for all nursing categories were inversely associated with operating margin. Specifically, higher hours per resident day (HPRD) for RNs (*β* = −3.66; 95% CI, −4.34 to −2.98; *p* < 0.001), LPNs (*β* = −2.67; 95% CI, −3.24 to −2.09; *p* < 0.001), and CNAs (*β* = −3.08; 95% CI, −3.42 to −2.75; *p* < 0.001) were associated with significantly lower margins. Ownership type also had a significant effect. Compared to for‐profit facilities, not‐for‐profit (*β* = −8.68; 95% CI, −9.42 to −7.95; *p* < 0.001) and government‐owned facilities (*β* = −8.05; 95% CI, −9.10 to −6.99; *p* < 0.001) had markedly lower operating margin. Higher occupancy rates (*β* = 0.02; 95% CI, 0.01–0.02; *p* < 0.001) and larger facility size measured by resident count (*β* = 0.11; 95% CI, 0.10–0.11; *p* < 0.001) were both positively associated with higher operating margins.

**TABLE 2:** Regression results between geographical location (rural vs. urban) and operating margin (*N* = 66,056 nursing home‐year observations).

	Model 1 – Location			Model 2 – Organizational factors			Model 3 – Environmental factors			Model 4 – Organization and environmental factors			Model 5 – Organization and environmental factors and COVID‐19		
		95% confidence interval			95% confidence interval			95% confidence interval			95% confidence interval			95% confidence interval	

	Beta coefficient	Low	High	Beta coefficient	Low	High	Beta coefficient	Low	High	Beta coefficient	Low	High	Beta coefficient	Low	High
***Independent variable***															
Location															
Urban	Ref	Ref	Ref	Ref	Ref	Ref	Ref	Ref	Ref	Ref	Ref	Ref	Ref	Ref	Ref
Rural	−2.19^**^	−3.77	1.01	−072	−0.44	2.46	−2.59^**^	−4.27	−0.92	−0.52	−2.03	0.99	−0.75	−2.29	0.79
***Organizational‐level factors***															
Star rating															
1				Ref	Ref	Ref				Ref	Ref	Ref	Ref	Ref	Ref
2				0.73^**^	0.22	1.23				0.75^**^	0.24	1.25	0.75^**^	0.25	1.26
3				1.41^***^	0.89	1.92				1.42^***^	0.91	1.94	1.45^***^	0.93	1.96
4				1.80^***^	1.27	2.33				1.81^***^	1.28	2.34	1.84^***^	1.31	2.37
5				2.15^***^	1.59	2.70				2.16^***^	1.61	2.71	2.20^***^	1.64	2.75
Nursing staff hours PRD															
RN				−3.66^***^	−4.34	−2.98				−3.26^***^	−3.94	−2.58	−3.20^***^	−3.88	−2.52
LPN				−2.67^***^	−3.24	−2.09				−2.17^***^	−2.75	−1.60	−2.14^***^	−2.72	−1.57
CNA				−3.08^***^	−3.42	−2.75				−3.04^***^	−3.38	−2.71	−3.05^***^	−3.38	−2.71
Chain affiliated															
No				Ref	Ref	Ref				Ref	Ref	Ref	Ref	Ref	Ref
Yes				1.68^***^	1.32	2.03				1.67^***^	1.32	2.03	1.67^***^	1.32	2.02
Ownership status															
For‐profit				Ref	Ref	Ref				Ref	Ref	Ref	Ref	Ref	Ref
Not‐for‐profit				−8.68^***^	−9.42	−7.95				−8.77^***^	−9.50	−8.03	−8.76^***^	−9.50	−8.03
Government				−8.05^***^	−9.10	−6.99				−7.85^***^	−8.91	−6.79	−7.86^***^	−8.92	−6.81
Occupancy rate				0.02^***^	0.01	0.02				0.01^***^	0.01	0.02	0.01^***^	0.01	0.02
Size (resident count)				0.11^***^	0.10	0.11				0.16^***^	0.15	0.16	0.16^***^	0.15	0.17
Payer mix															
Private				Ref	Ref	Ref				Ref	Ref	Ref	Ref	Ref	Ref
Medicare				0.19^***^	0.17	0.20				0.18^***^	0.17	0.20	0.19^***^	0.17	0.20
Medicaid				0.04^***^	0.03	0.05				0.05^***^	0.04	0.05	0.05^***^	0.04	0.05
Resident complexity				0.18	−0.10	0.46				0.26	−0.02	0.54	0.27	−0.00	0.55
Resident racial/ethnic mix															
White				Ref	Ref	Ref				Ref	Ref	Ref	Ref	Ref	Ref
Black				0.00	−0.02	0.02				0.01	−0.01	0.03	0.01	−0.01	0.03
Asian				0.02	−0.03	0.07				0.04	−0.01	0.09	0.04	−0.01	0.09
Hispanic				0.05^***^	0.02	0.08				0.06^***^	0.03	0.08	0.05^***^	0.03	0.08
Other race				−0.05^***^	−0.06	−0.03				−0.04^***^	−0.05	−0.03	−0.04^***^	−0.05	−0.03
***Environmental‐level factors***															
Competition (1 − HHI)							0.01	−0.01	0.02	−0.01	−0.02	0.01	0.01	−0.02	0.02
Population 65 and over							−0.01	−0.11	0.09	0.09	−0.01	0.18	0.08	−0.01	0.18
Household income							−0.01^***^	0.01	0.01	−0.01^***^	0.01	0.01	−0.01^**^	0.01	0.01
Medicare advantage penetration							−0.01	−0.04	0.02	−0.02	−0.04	0.01	−0.01	−0.03	0.03
***COVID‐19***															
Pre‐COVID													Ref	Ref	Ref
COVID (2020–2022)													−9.37^***^	−9.79	−8.95
***COVID‐19 interaction with location***															
Urban^*^COVID‐19													Ref	Ref	Ref
Rural^*^COVID‐19													−1.18^***^	−1.58	−0.78

Abbreviations: CNA, certified nurse assistant; HHI, Herfindahl–Hirschman index; LPN, license practical nurse; PRD, per resident day; RN, registered nurse.

*** *p <* 0.001.

** *p <* 0.01.

* *p <* 0.05.

**FIGURE 2:**
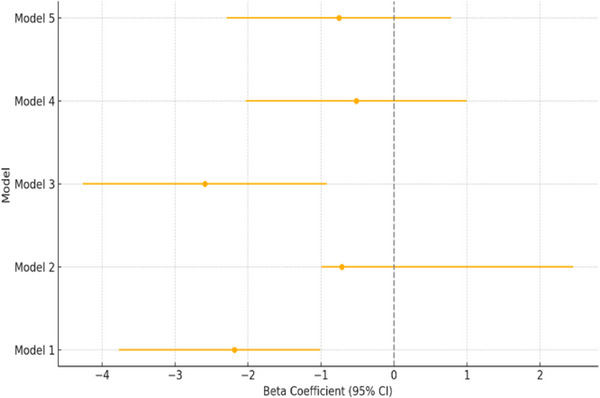
Rural versus urban operating margin summary across models. CI, confidence interval. *Note*: Model 1 = Rural location only; Model 2 = Location and organizational factors; Model 3 = Location and environmental factors; Model 4 = Location, organizational, and environmental factors; Model 5 = Location, organizational, and environmental factors, and COVID‐19.

In Model 3, which adjusted for environmental factors only, rural nursing homes had 2.59% lower operating margin compared to urban nursing homes (*β* = −2.59; 95% CI, −4.27 to −0.92; *p* < 0.01), or 0.4% lower operating margin compared to the unadjusted model. Higher household income was negatively associated with operating margin (*β* = −0.01; 95% CI, 0.01–0.01; *p* < 0.001). In Model 4, after controlling for both organizational and environmental factors, the association between rural location and operating margin was no longer statistically significant (*β* = −0.52; 95% CI, −2.03 to 0.99; *p* > 0.05). Finally, in Model 5, which accounted for organizational and environmental factors as well as the impact of COVID‐19, rural location was not significantly associated with operating margin (*β* = −0.75; 95% CI, −2.29 to 0.79; *p* > 0.05). However, COVID‐19 was associated with a significant overall reduction in operating margins (*β* = −9.37; 95% CI, −9.79 to −8.95; *p* < 0.001). In addition, the interaction term between the COVID‐19 indicator and rural facilities suggest that rural nursing homes experienced a more negative effect on operating margin due to COVID‐19 compared to urban nursing homes (*β* = −1.18; 95% CI, −1.58 to −0.78; *p* < 0.01). Figure 2 includes a forest plot, which depicts the changes in operating margin as we move from Model 1 to Model 5.

## DISCUSSION

This study examined the financial performance differences between rural and urban facilities and explored the role that organizational and environmental factors may play in explaining these differences. In addition, we explored how COVID‐19 may have contributed to these financial differences. Operating margin was used to compare the financial performance of these facilities.

The unadjusted findings (Model 1) show that rural nursing homes have lower average operating margin compared to urban facilities, in alignment with the prior research.^7^ These results suggest the significant financial challenges for rural facilities and highlight the structural challenges faced by these facilities.^10^, ^33^, ^34^ However, the operating margin disparity between rural and urban nursing homes disappears after accounting for organizational factors such as size, occupancy rates, and payer mix in the adjusted regression model (Model 2). This suggests that organizational factors account for much of the variation in the lower operating margin of rural nursing homes.

Regarding size, our results show that rural facilities have significantly fewer residents compared to urban facilities. This smaller scale may prevent rural facilities from achieving economies of scale, as they may not be able to spread fixed costs—such as staffing, utilities, and maintenance—across as many residents as larger urban facilities.^3^, ^22^ Consequently, fixed costs per resident may be higher, which may lead to reduced profitability among rural nursing homes.^3^, ^22^ Similarly, lower occupancy rate may contribute to the differences in financial performance, especially in rural nursing homes. A lower occupancy rate makes it difficult for rural nursing homes to operate efficiently,^1^, ^11^ as revenue per available bed decreases while operating costs stay relatively constant, increasing financial strain.^17^, ^19^, ^25^, ^35^, ^36^ Rural nursing homes also have a higher percentage of Medicaid payer mix, which may contribute to the differences in financial performance. Nursing homes with a higher percentage of Medicaid residents may be at a financial disadvantage because Medicaid reimbursement rates are typically lower than those of Medicare and private payers.^12^, ^37^

Rural nursing homes also report lower nurse staffing ratios and lower quality. The lower nurse staffing ratios in rural facilities may be driven by a lack of available workers and budgetary limitations that may make it more difficult to recruit and retain skilled employees.^7^, ^9^, ^33^, ^34^, ^35^ The combination of lower quality and staffing ratios suggests that rural nursing homes face systemic difficulties providing high‐quality care, which could further hinder their capacity to compete in the long‐term care market and maintain their financial stability.^7^, ^8^, ^34^

In terms of organizational characteristics that influenced operating margins in our adjusted (Model 2), several had notable effects. For instance, facilities with higher CMS star ratings had better financial performance, suggesting that quality and profitability are aligned—possibly due to increased demand or operational efficiency.^4^, ^6^, ^12^ Facilities owned by a chain also had higher operating margins, reflecting potential benefits from centralized resources or shared administrative services.^6^, ^9^ While higher staffing levels for RNs, LPNs, and CNAs were associated with lower margins, this may reflect increased labor costs, which are not always offset by reimbursement rates.^4^, ^6^, ^12^ For‐profit ownership, higher occupancy, and larger facility size had higher operating margin, underscoring the value of operational scale and efficient capacity use.^9^, ^12^ In addition, facilities with higher proportions of Medicare and Medicaid residents were associated with higher operating margins compared to those with more private‐pay residents. One possible explanation for this finding—particularly in the case of Medicaid—is the influence of state‐specific Medicaid policies. Although we control for state fixed effects, this approach does not capture time‐varying policy changes at the state level. Other contributing factors may include the predictability of Medicaid payments and the operational efficiencies developed by high‐Medicaid providers.^12^ The positive association between Medicare share and operating margin is consistent with Medicare's relatively higher reimbursement rates.^12^, ^19^ Together, these findings suggest that the relationship between payer mix and operating margin is more complex than typically assumed and should be interpreted in the context of broader market dynamics and facility‐level strategies.

Our study findings also showed that rural nursing homes had substantially lower operating margins than urban facilities when only environmental factors were considered (Model 3). This suggests that environmental factors play a role in exacerbating the financial challenges faced by rural nursing homes. Rural nursing homes serve a larger proportion of the older population, who need more acute and complex care, which may potentially increase operational costs and increase the financial strain of these facilities.^38^, ^39^ Additionally, rural nursing homes are often located in areas with a lower household income, typically serving poorer communities.^1^, ^11^, ^40^ These communities may have a higher rate of Medicaid reliance, which could further diminish the revenue streams.^40^, ^41^, ^42^ Our adjusted (Model 3) showed that household income at the county level was negatively associated with operating margin, which may reflect higher labor and operational costs in more affluent markets or increased competition from alternative care options, such as assisted living facilities and home‐based services.^43^

It is important to highlight that while both organizational and environmental factors are important, our findings indicate they play distinct roles in shaping rural nursing homes’ financial performance. Model 2 shows that after adjusting for organizational characteristics—such as size, occupancy, ownership, and staffing—the rural–urban gap in operating margin disappears, suggesting that these internal operational characteristics largely account for observed differences. In contrast, Model 3 shows that even after accounting for environmental factors such as household income or competition, rural nursing homes still had lower operating margins suggesting that while environmental factors may compound financial challenges, they do not fully explain the observed disparities. Model 4 reinforces the combined effects of the second and third model that organizational and environmental factors interact to shape nursing homes financial performance. Improving rural nursing homes’ financial viability may require organizational restructuring and investment in operational efficiency—alongside broader policy efforts aimed at addressing the systemic challenges unique to rural facilities.

The inclusion of the COVID‐19 variable (Model 5) provided further insights into the financial vulnerabilities of rural nursing homes. Our results suggest that rural nursing homes experienced a disproportionately greater decline in operating margins compared to their urban counterparts during the pandemic. This amplified impact highlights the compounded challenges faced by rural facilities, such as increased operational costs, lower occupancy rates, and limited access to emergency resources.^3^, ^8^, ^10^, ^12^, ^34^, ^44^, ^45^

Our study has several limitations that merit attention. First, while there is increasing research on nursing homes potentially disguising profits via related‐party transactions,^25^ this aspect was not explored in our analysis. Second, our reliance on CMS Cost Reports could introduce discrepancies due to timing differences, as these reports are based on fiscal year data. Third, our analysis was based on secondary data from CMS Cost Reports, which are submitted to CMS by the facilities themselves and are not subject to regular audits, potentially impacting the data accuracy. Lastly, though our study included state‐fixed effects, it is worth noting that states have their own diverse regulatory and economic environment that may influence nursing home operations, but may not have been considered in this study.

Despite our study's limitations, our study offers useful policy insights. Considering that the rural–urban difference is nonsignificant after controlling for organizational factors indicates that structural challenges, such as smaller size, lower occupancy, and a greater reliance on Medicaid rather than geographic location, may contribute to the lower profitability of rural nursing homes. Policymakers should prioritize interventions that address organizational constraints by supporting strategies such as increasing Medicaid reimbursement rates, promoting regional partnerships to improve economies of scale, and expanding marketing outreach efforts to attract residents from neighboring communities to increase occupancy in rural nursing homes.^15^, ^46^, ^47^

If increasing Medicaid reimbursement rates is not feasible, policymakers should consider supplemental funding for nursing homes with a high proportion of Medicaid residents. Additionally, offering subsidies or grants for efficiency improvements, technology adoption, and quality initiatives can foster innovation in low‐competition areas. For nursing home management and administrators, insights from this study can be valuable to refine their operational strategies. Nursing home administrators must effectively balance economic efficiency with high care standards. The financial stability of nursing homes will increasingly rely on their ability to navigate the changing reimbursement environment and operational challenges. One of the most pertinent examples is the proposed expansion of the value‐based purchasing (VBP) in nursing homes, expected to take effect in 2026 that will tie Medicare reimbursement to a number of quality metrics, encouraging nursing homes to improve care quality in order to receive larger reimbursement.^48^ Understanding how specific state policies and economic conditions influence nursing home operations and financial outcomes could lead to more tailored and effective policy recommendations.

## CONCLUSIONS

This study provides important insights into the financial disparities between rural and urban nursing homes, shedding light on the organizational and environmental factors that influence financial outcomes. By highlighting these differences, this research underscores the need for targeted policy interventions that support the financial sustainability of nursing homes, particularly in rural areas. As the demand for long‐term care services continues to grow, ensuring the financial health of these facilities is essential for providing quality care to the nation's aging population.

## CONFLICT OF INTEREST STATEMENT

The authors declare no conflicts of interest.
